# Alleviating liver failure conditions using an integrated hybrid cryogel based cellular bioreactor as a bioartificial liver support

**DOI:** 10.1038/srep40323

**Published:** 2017-01-12

**Authors:** Apeksha Damania, Mohsin Hassan, Nana Shirakigawa, Hiroshi Mizumoto, Anupam Kumar, Shiv K. Sarin, Hiroyuki Ijima, Masamichi Kamihira, Ashok Kumar

**Affiliations:** 1 Department of Biological Sciences and Bioengineering, Indian Institute of Technology Kanpur, Kanpur-208016, UP, India; 2 Institute of Liver and Biliary Sciences, New Delhi, India; 3 Department of Chemical Engineering, Faculty of Engineering, Kyushu University, Fukuoka- 8190395, Japan

## Abstract

Conventionally, some bioartificial liver devices are used with separate plasmapheresis unit to separate out plasma from whole blood and adsorbent column to detoxify plasma before it passes through a hepatocytes-laden bioreactor. We aim to develop a hybrid bioreactor that integrates the separate modules in one compact design improving the efficacy of the cryogel based bioreactor as a bioartificial liver support. A plasma separation membrane and an activated carbon cloth are placed over a HepG2-loaded cryogel scaffold in a three-chambered bioreactor design. This bioreactor is consequently connected extracorporeally to a rat model of acute liver failure for 3 h and major biochemical parameters studied. Bilirubin and aspartate transaminase showed a percentage decrease of 20–60% in the integrated bioreactor as opposed to 5–15% in the conventional setup. Urea and ammonia levels which showed negligible change in the conventional setup increase (40%) and decrease (18%), respectively in the integrated system. Also, an overall increase of 5% in human albumin in rat plasma indicated bioreactor functionality in terms of synthetic functions. These results were corroborated by offline evaluation of patient plasma. Hence, integrating the plasmapheresis and adsorbent units with the bioreactor module in one compact design improves the efficacy of the bioartificial liver device.

Extracorporeal liver devices are considered the next best alternative to liver transplantation by aiding in the recovery of patients to normal health in the case of acute liver failure (ALF) or returning them to a state before decompensation in the case of acute-on-chronic liver failure (ACLF)^1^,^2^. These extracorporeal support systems, aimed at improving the transplant-free survival of patients, may also serve as a bridge to transplantation and/or prevent progress to further severe damage to the liver and other vital organs that could result in end-organ dysfunction^1^,^3^,^4^,^5^,^6^.

An ideal extracorporeal liver device should restore both synthetic as well as detoxification functions of the liver. Artificial liver devices centered on the detoxification function of the liver, use selective membranes and adsorbents based on the principle of adsorption and physical/chemical gradients^7^,^8^ to remove putative toxins associated with liver failure. Removal of toxins alone by these artificial liver devices may allow for the recovery of metabolic functions but without any additional synthetic activity^9^,^10^. Randomized clinical trials using the Molecular Adsorbent Recirculating System (MARS) have shown significant dialysis effects with a considerable reduction in serum ammonia, bilirubin and bile acids along with a slight improvement in hepatic encephalopathy. However, all these large clinical trials have failed to demonstrate any survival benefit for the patients treated with MARS^11^,^12^,^13^.

Bioartificial liver (BAL) devices are based on the inclusion of a biological component like primary liver cells to replace to a large extent some of the major synthetic functions of the liver^7^,^14^,^15^. However, a purely biological device may not achieve synthetic and detoxification functions to its full potential^2^.

An integrated unit combining artificial and bioartificial device modules may help fulfill synthetic as well as detoxification roles of the liver to its full capacity. Taking cue from previously used charcoal hemoperfusion systems for treatment of ALF patients, some BAL devices are connected to a separate adsorbent column (typically activated charcoal) to remove some toxins in the plasma before it passes through a cell-loaded bioreactor^16^,^17^. In recent years, hybrid BAL devices have been explored that combine the detoxifying capacity of an adsorbent column with the synthetic function of a cell-loaded bioreactor^18^,^19^ with positive outcomes in terms of both safety and efficiency. Conventionally, a plasmapheresis device that separates out the plasma component from the circulating blood is also used along with the BAL device.

Recently our work showed the potential of poly(*N*-isopropylacrylamide)-chitosan (poly(NiPAAm)-chitosan) cryogel as a hepatocyte carrier for bioartificial liver support^20^. Cryogels, due to their inherent interconnected porosity, mimic the capillary network of hollow fibre bioreactors (the most clinically accepted bioreactor design for BAL devices), providing a large surface area-to-volume ratio for the immobilization and culture of a large number of hepatocytes in a small volume^21^. Poly(NiPAAm)-chitosan cryogel continuous bioreactors showed the ability to maintain functionality of hepatocytes as well as detoxify circulating plasma obtained from ACLF patients^20^.

In this paper, a hybrid cryogel based bioreactor was designed that integrates separate bioreactor module, plasma separation module and activated charcoal module of conventional BAL setups into one compact device so as to improve the efficacy of the bioreactor and reduce the amount of blood withdrawn for circulation. This integrated unit was then connected to a rodent ALF model and liver functions analyzed. The results were compared to those of a conventional cryogel bioreactor setup to highlight the importance of integrating the separate modules.

## Materials and Methods

### Synthesis of poly(*N*-isopropylacrylamide)-chitosan cryogels

Poly(*N*-isopropylacrylamide)-chitosan cryogels were synthesized using a method previously described^20^. A solution of 2% chitosan (low viscosity, Sigma, USA) was prepared in 5 mL of 0.1 M acetic acid. Solutions of *N*-isopropylacrylamide (Acros Organic, NJ, USA) and methyl-bisacrylamide (MBAAm) (Sisco Research Labs, Mumbai, India) (NiPAAm:MBAAm ratio 3:1) were prepared in 4 mL degassed water. Both the solutions were mixed and ammonium persulphate (APS) (0.2% w/v) (Merck, Mumbai, India) added. Further glutaraldehyde (chitosan:glutaraldehyde ratio 15:1) (Loba Chemie, Mumbai, India) and *N*,*N*,*N*’,*N*’-tetramethylethylenediamine (TEMED) (Loba Chemie, Mumbai, India) (0.15% w/v) were added and mixed thoroughly. The solutions were immediately poured in appropriate moulds (2.5 mL syringes or non-treated 6 well plates) and frozen at −12 °C for 16 h. Post incubation, cryogels were thawed at room temperature and washed with distilled water followed by air drying.

### Culture and seeding of HepG2 cells

HepG2 cells were obtained from National Centre for Cell Science (NCCS), Pune, India. The cells were cultured in Dulbecco’s Modified Eagles’ Medium (DMEM) (Sigma, USA) supplemented with 10% fetal bovine serum (FBS) (Invitrogen, USA) and 1% antibiotic (HiMEDIA, India) with the pH maintained at 7.4, in a 37 °C humidified incubator with 5% CO_2_. Media was changed every alternate day and cells passaged at 70% confluency. For cell seeding, both monolith (height 40 mm; diameter 8 mm) and disc-shaped (height 3 mm; diameter 35 mm) cryogels were sterilized in 70% ethanol, followed by washing in phosphate buffer supplemented with 2% antibiotic. HepG2 cells were seeded into the cryogels (1.5 × 10^6^ cells per scaffold) using a perfusion based system as described earlier^20^. Five-day cell-seeded cryogels were used for further animal and *in vitro* studies.

### Developing the integrated hybrid bioreactor design

**Figure 1:**
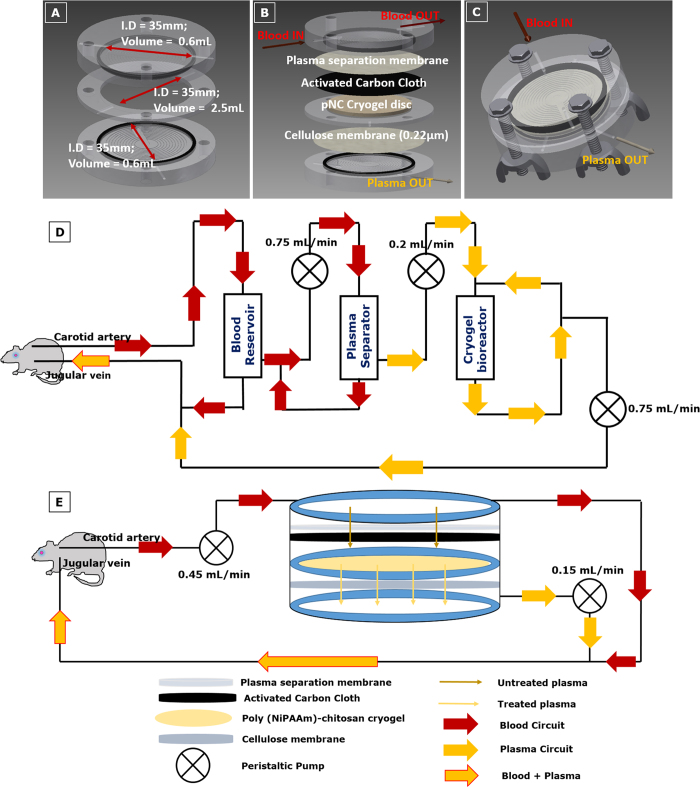
Integrated hybrid cryogel-based bioreactor design. (**A**) Dimensions of the bioreactor; (**B**) Constituents of the three compartments; (**C**) Compact bioreactor design with chambers clubbed together; Schematic representation of conventional extracorporeal BAL (System 1) with separate plasma separation module (**D**) and of the hybrid cryogel based bioreactor system (System 2) integrating the plasmapheresis device and adsorbent column module with the bioreactor module (**E**).

A three-chambered integrated hybrid bioreactor was designed by combining the plasmapheresis module and adsorbent module together with the bioreactor module. Three disc shaped units were placed such that the top unit consists of a plasma separation membrane (Vivid^TM^ Plasma Separation Membrane, Pall Life Sciences, USA) placed over an activated charcoal cloth (a gift from Prof. Nishith Verma, Department of Chemical Engineering, Indian Institute of Technology Kanpur, India); the middle unit contains the hepatocyte seeded cryogel disc; and the lower unit contains a 0.22 μm cellulose filter (Millipore, India). Each chamber is connected to the other and has an inlet and outlet to allow for exchange of nutrients as well as unhindered circulation of blood and plasma. The total volume of the entire system is approximately 4 mL (Fig. 1A–C).

#### Studying the adsorption capacity of activated carbon cloth

Activated carbon cloth was cut into discs of 20 mm diameter and placed in a smaller reactor unit. Plasma separation membrane, cryogel disc and cellulose membrane were removed from the reactor for this study. Plasma collected from rats 1–2 h post induction of liver injury was circulated offline in the reactor containing the activated carbon cloth for 3 h at a flow rate of 0.2 mL/min. At the end of the 3 h, plasma was analyzed for amount of bilirubin, albumin, ammonia, urea and aspartate transaminase (AST) using a biochemical analyzer (ERBA Mannheim, Germany).

#### Effect of pre-treatment with activated carbon on cell viability and functionality in presence of ALF plasma

To check the effect of pre-treatment with activated carbon cloth, plasma collected from ALF rats 1–2 h post induction of injury, was first perfused through activated carbon cloth as described earlier and then perfused for 3 h at a flow rate of 0.2 mL/min through a 5-day cell-seeded cryogel bioreactor (3D) or incubated for 3 h with cells cultured in monolayer (2D) (density 5 × 10^4^ cells/well). Viability of the cells was measured using 3-(4,5-dimethylthiazol-2-yl)-2,5-diphenyltetrazolium bromide (MTT) (Sigma Aldrich, USA) assay and Lactate dehydrogenase (LDH) (Sigma Aldrich, USA) activity before and after treatment with plasma. Absorbance values from the MTT assay were normalized to cell number using a standard curve. Functionality in terms of albumin synthesis was measured before and after treatment with ALF plasma using standard sandwich ELISA as per manufacturer’s protocol (Bethyl Laboratories, USA). For microscopic analysis on the effect of ALF plasma, cells were fixed using 4% paraformaldehyde followed by staining with the nuclear stain, 4′,6-diamidino-2-phenylindole (DAPI) (Sigma Aldrich, USA).

### Rat model for acute liver failure

Male Wistar rats (250–350 g) were used for development of an animal model for ALF as per the approval of Institute Animal Ethics Committee (IITK/IAEC/2014/1022) and all methods were performed in accordance with the relevant guidelines and regulations of this committee. The animals were housed in climate-controlled environment with alternate 12 h light and dark cycles and standard food and water provided. Partial hepatectomy (2/3^rd^) was performed according to the method described by Higgins and Anderson with some modifications. A mid-abdominal incision was made and the upper abdomen and lateral lower portions of both hemithoraces compressed to partly exteriorize the liver. Further a 2–0 cotton thread was used to tie down the medial and left lateral lobes close to the hilum. Consequently, the hepatic portal vein and hepatic artery were clamped for 40 minutes to create an ischemic condition. The knotted lobes were then resected *en bloc* and the abdomen sutured closed using prolene sutures.

### Extracorporeal BAL system

In the first system (System 1) we used a modified version of the extracorporeal BAL system used by Flendrig *et al*.^22^. Plasmapheresis modules were made (length 50 mm; internal diameter 8 mm; internal volume 2.5 cm^3^) using plasma separation membrane capillaries (internal diameter of 330 μm; surface of 0.5 m^2^) (PlasmaFlo, Japan) stacked tightly together. A blood reservoir was introduced to help prevent blood loss and maintain pressure differences due to different flow rates (Fig. 1D).

In the second system (System 2), the integrated hybrid unit was connected directly to the animal as shown in the schematic (Fig. 1E). The flow rates were optimized to enable maximum plasma separation.

Both the systems were primed using heparinized phosphate buffer followed by healthy rat plasma. In addition, heparin was used as an anticoagulant as well during the experiment at a dose of 500 IU/h. In both systems, the rat was infused with dextrose and saline at regular time intervals to prevent hypoglycemia and dehydration.

In both System 1 and 2, animals were divided into two major groups (n = 8 per group; n = 4 per sub-group), namely the experimental group and the control group (constituting healthy rats (negative control) and ALF model rats (positive control)). In System 1, the experimental group constituted of healthy rats and ALF model rats both connected to the bioreactor. In System 2, the experimental group consisted of ALF model rats and healthy rats connected to cell-seeded bioreactor and ALF model rats connected to acellular bioreactor.

The animals were connected to the bioreactor setup 1–2 h post-induction of injury and plasma samples (0.2–0.5 mL) collected at known time intervals. Plasma was analyzed for liver function parameters using a blood biochemical analyzer (ERBA Mannheim, Germany). Further, human albumin was measured in the plasma samples using sandwich ELISA as per standard protocol.

Used cryogel bioreactors were fixed using 2.5% glutaraldehyde for 6 h at 4 °C. After incubation, gradient wash using varying concentrations of ethanol was given and the samples vacuum dried. Cross-sections of the cryogels were analyzed using scanning electron microscopy (SEM) and compared against unused five-day cell-seeded cryogel bioreactors.

### Offline evaluation of integrated cryogel based bioreactor

Plasma from alcoholic ALF patients taken for routine clinical examination was used for offline evaluation of the integrated bioreactor. Informed consent was obtained from each of the patients prior to the experiments. The methods followed were according to guidelines and regulations of the human ethics and safety committee of the Institute of Liver and Biliary Sciences, New Delhi, India. The experimental protocol involving humans was approved by the human ethics and safety committee of the Institute of Liver and Biliary Sciences, New Delhi, India (IEC/IRB no: 21/6 dated 26/06/2013). Five-day HepG2 loaded cryogel bioreactor was washed with PBS and plasma from 9 ALF patients circulated on 9 independent cryogel bioreactors for 3 h. Untreated and treated plasma was quantified for changes in liver function parameters using a biochemical analyzer.

### Statistical analysis

Statistical comparisons were evaluated using the GraphPad Prism software. One-way-ANOVA with repeated measures was used to analyze the data obtained from the *ex vivo* studies of both System 1 and System 2. Further, the differences between two groups were analyzed using the Student’s t- test.

## Results and Discussion

### Synthesis of poly(N-isopropylacrylamide)-chitosan cryogel

Poly(*N*-isopropylacrylamide)-chitosan cryogel was synthesized by the process of cryogelation. In this process, as described earlier^20^, incubation at sub-zero temperatures causes freezing of some of the solvent (water), resulting in cryoconcentration of the dissolved monomer/polymer precursors in the non-frozen liquid phase (NFLP). Gelation/cross-linking of the precursors takes place in the NFLP leading to the formation of thick pore walls around the frozen ice crystals. Upon thawing, ice crystals melt revealing an interconnected macroporous structure. The physio-chemical characterization of the poly(*N*-isopropylacrylamide)-chitosan cryogels has been carried out previously^20^ and the pore size of the cryogel was found to be in the range of 20–100 μm, with porosity of 87.6 ± 2.0% and a swelling ratio of 0.94 ± 0.001. In addition the resistance to liquid flow was found to be between 7 mL min^−1^ and 10 mL min^−1^ indicating a bicontinuous water and polymer phase with interconnected pores^20^ supporting the use of this cryogel as a potential bioreactor matrix.

### Designing an integrated hybrid bioartificial liver bioreactor unit

Size of the bioreactor is critical for BAL, since, the bigger the bioreactor, the larger the volume of blood withdrawn from the patient and only a limited amount of blood/plasma may be taken out of a patient at any time^23^. Also, the more the number of modules connected to the reactor, the more the volume of blood withdrawn. Hence, an integrated system was designed to reduce the amount of blood withdrawn, by clubbing three separate modules into one unit. For this, a polysulfone membrane that permits plasma separation in lateral flow was placed in direct contact with an activated carbon cloth that acts as a receiving medium for the separated out plasma. This chamber containing the plasmapheresis and adsorbent module was then placed over the chamber containing the cell-laden cryogel disc which was further placed over a cellulose membrane containing lower chamber. A cellulose membrane was introduced in the design to entrap any loose hepatocytes that may leach off the cryogel matrix and enter into circulation. Treated plasma from the lower chamber combines with the blood circuit from the top-most chamber.

#### Studying adsorption capacity of activated carbon cloth

Liver failure is associated with a large number of water soluble and protein bound toxins^24^, such as ammonia and bilirubin whose levels increase almost four-fold in ALF^25^. Inclusion of an adsorbent column such as activated carbon in a BAL device helps reduce the overall toxin load on the bioreactor since it adsorbs some of the toxins that could potentially damage the cells housed in the bioreactor^24^,^26^,^27^.

**Figure 2:**
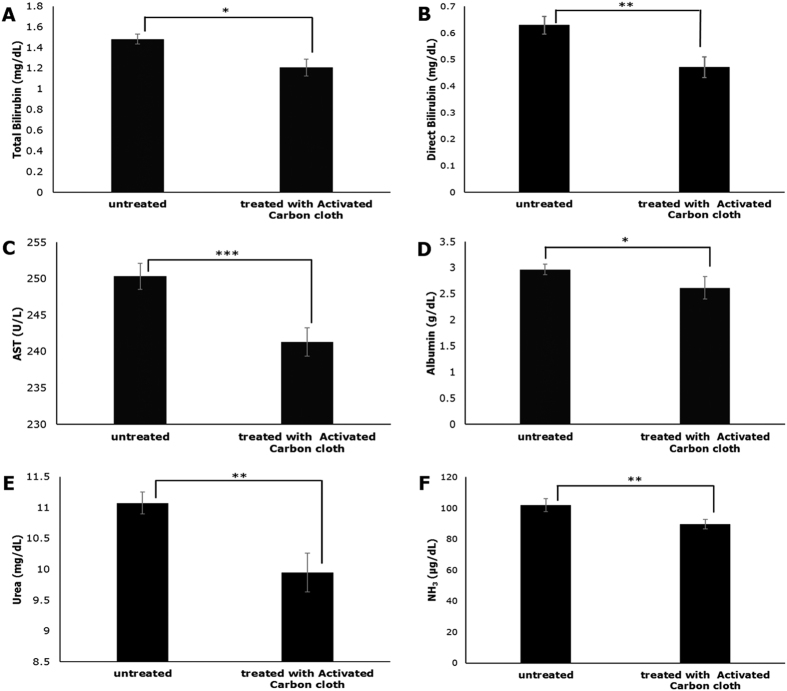
Adsorption capacity of activated carbon cloth. Adsorption of bilirubin (total and direct), (**A**) and (**B**), AST (**C**), albumin (**D**), urea (**E**) and ammonia (**F**) by the activated carbon cloth after perfusion with plasma from a rat model with acute liver failure (n = 3, *p < 0.05, **p < 0.01, ***p < 0.001).

Activated carbon cloth was treated with plasma from ALF rodent model to study its capacity to adsorb toxins associated with liver failure. A significant amount of toxins was found to be adsorbed by the activated carbon cloth (Fig. 2). Approximately 10–20% of direct bilirubin, urea and ammonia was adsorbed, showing efficacy of activated carbon cloth in removal of some of the toxins associated with liver failure.

#### Effect of plasma on viability of cell seeded in bioreactor

ALF plasma contains high levels of hepatotoxic compounds such as cytokines, lipopolysaccharides and biomolecules that are normally detoxified by the liver but due to impaired liver function tend to accumulate in the plasma like ammonia and bile acids^28^. All these toxins could exert a detrimental effect on the cells used within a BAL device, such as loss of cell-cell contact, cell death due to apoptosis and necrosis, and loss in functionality^28^,^29^,^30^.

**Figure 3:**
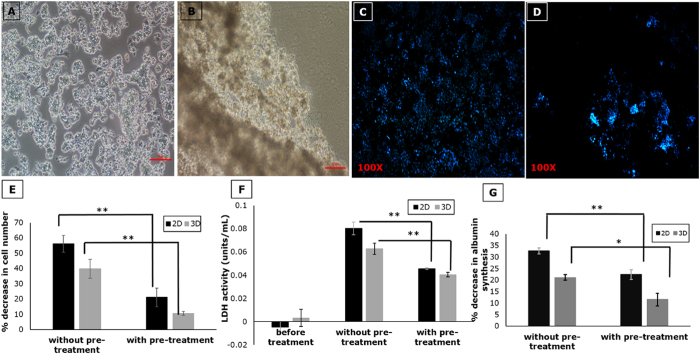
Effect of pre-treatment of ALF plasma with activated carbon cloth on cell-seeded bioreactor. Phase contrast image of hepatocytes before (**A**) and after (**B**) treatment with rat ALF plasma showing loss of cell-cell contact (Scale bar 10 μm); DAPI staining before (**C**) and after (**D**) treatment with rat ALF plasma illustrating cell loss; (**E**) Percentage decrease in cell number; (**F**) LDH activity and (**G**) Percentage decrease in albumin synthesis in 2D and 3D before and after pre-treatment with activated carbon cloth. (n = 3, *p < 0.05, **p < 0.01).

Microscopic analysis of hepatic cells in the presence of ALF model plasma showed significant cell loss (Fig. 3A–D). Viability and functionality of hepatocytes in 2D culture and 3D scaffolds was studied with and without pre-treatment of the plasma with activated carbon cloth. It was observed that pre-treatment of plasma with activated carbon resulted in a significantly lower percentage decrease in cell number (16–20%) as compared to cells exposed to ALF plasma without pre-treatment (40–55%) (Fig. 3E). Further, LDH activity of the cells measured before and after treatment with plasma showed a significant decrease on pre-treatment of plasma with activated carbon indicating a decrease in cytotoxicity (Fig. 3F). Albumin synthesis also showed similar results (Fig. 3G), confirming that pre-treatment of plasma with activated carbon reduces the toxic load faced by the cell-seeded bioreactor.

### Animal study using the extracorporeal BAL setup

One of the most popular and classic models of liver failure is the two-thirds (70%) partial hepatectomy, first established by Higgins and Anderson^31^. Liver resections are well tolerated in rodents with minimal operative mortality. The classical technique of ligature *en bloc* carries a high risk of injuries and may compromise other lobes. Also, liver resections are associated with a lot of intraoperative blood loss. Hence, to overcome these problems, Pringle^32^ introduced the Pringle maneuver technique in which the portal vein and the hepatic artery are clamped before resection of the lobes to reduce the risk of bleeding from the stump and simultaneously induce ischemic injury^33^,^34^,^35^. In our model, before resection, the portal vein and hepatic artery were clamped for 40–45 minutes to induce maximum ischemic injury. These ALF models were connected to the bioreactor setup 1–2 hours post injury induction to allow for the effects of injury to set in. Biochemical parameters typically used in clinical evaluation of liver function were analyzed at predetermined time intervals for 3 hours after connection to the extracorporeal BAL system. A treatment regime of 3 hours was selected to be able to make an effective comparison between the two systems keeping in mind two major limiting factors: (i) the stability of the animals with respect to performance, which tends to decrease and/or vary unpredictably beyond 3 hours due to stress caused by the extensive invasive surgery involved in inducing the liver failure as well as connecting the animal to an extracorporeal device and (ii) the reduced efficiency of the bioreactor in System 1 beyond 3 hours which can be attributed to cell death resulting from exposure to toxins in the plasma as observed in our previous work^20^.

**Figure 4:**
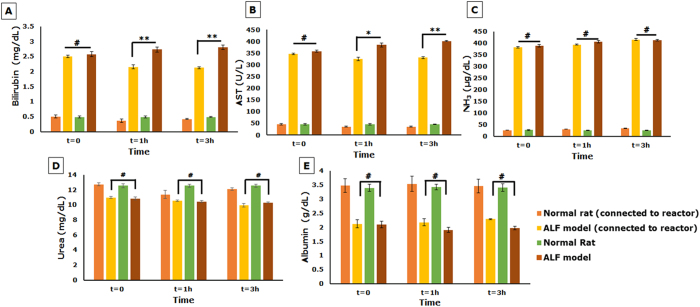
Biochemical analysis of efficacy of cryogel bioreactor in System 1. (**A–E**) Liver function parameters analyzed at predetermined time intervals; Statistical analysis using one-way ANOVA followed by Student’s t-test where n = 4, ^#^Non-significant, *p < 0.05 and **p < 0.01.

In System 1, though there was a significant difference (p < 0.01 for bilirubin and p < 0.05 for AST, by one-way ANOVA followed by Student t-test) in some of the parameters of the rat model connected to the bioreactor as compared to the untreated rat model, the values after the first hour of connection remained unchanged (Fig. 4). Bilirubin and AST decreased within the first hour of connection, beyond which there was no further decline (Fig. 4A,B). Additionally, ammonia levels of both rat models connected to bioreactor and left untreated showed a gradual increase though one-way ANOVA revealed no statistically significant difference between the ALF model connected to the bioreactor and the untreated ALF model (Fig. 4C). Further, levels of urea and albumin remained constant in both the model connected to the bioreactor and untreated model (Fig. 4D,E) with no statistically significant difference between the two as revealed by one-way ANOVA. These results could be attributed to the fact that the high concentration of toxins present in the plasma may have caused a detrimental effect on the cells housed in the bioreactor unit resulting in loss of functionality.

**Figure 5:**
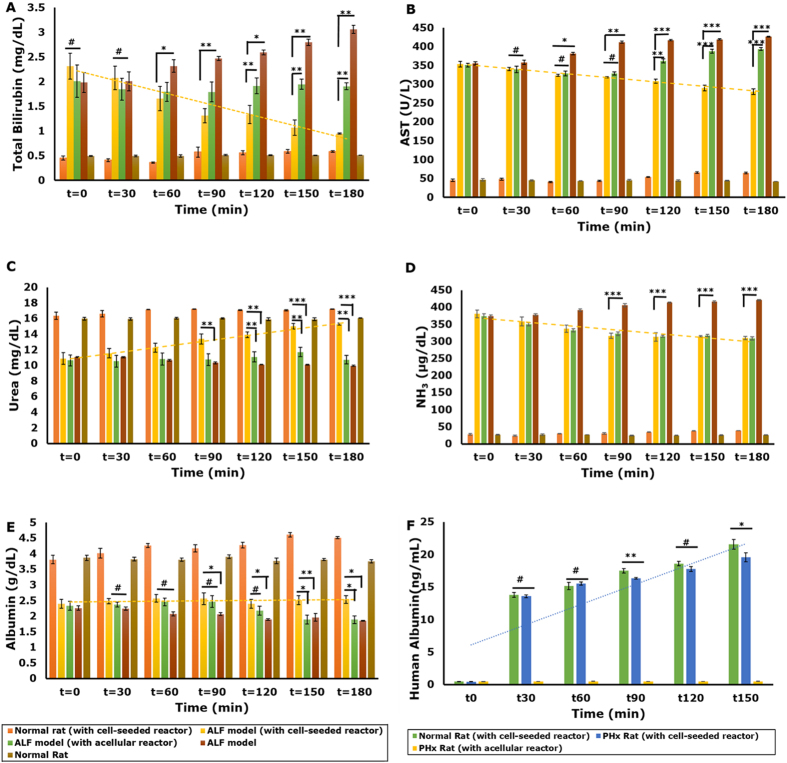
Biochemical analysis of efficacy of integrated hybrid cryogel based bioreactor in System 2. Liver function parameters Bilirubin (**A**), AST (**B**), Urea (**C**) Ammonia (**D**) and Albumin (**E**) analyzed at predetermined time intervals after extracorporeal connection; (**F**) Quantification of human albumin in rat plasma. (Statistical analysis using one-way ANOVA with repeated measures and Student’s t-test where n = 4; ^#^Non-significant, *p < 0.05, **p < 0.01 and ***p < 0.001).

The integrated hybrid bioreactor design aims at not only reducing the overall toxic burden on the bioreactor unit, but also developing a more compact system that minimizes the amount of blood withdrawn from a patient for extracorporeal circulation. In System 2, a one-way repeated measures ANOVA was run on plasma samples of four ALF models to determine if there were differences in concentration of the various biochemical parameters for liver function over the duration of the treatment. The results showed that the treatment with the integrated bioreactor elicited statistically significant differences in the mean values of the biochemical parameters over the time course, bilirubin, F = 49.1, p < 0.001, AST, F = 383.2, p < 0.0001, urea, F = 87.46, p < 0.0001, ammonia, F = 718.5, p < 0.0001 and albumin, F = 180.4, p < 0.0001. In addition, a significant difference was observed in the biochemical parameters between models connected to a cell-seeded bioreactor and untreated models (p < 0.01 for albumin and p < 0.05 for AST, bilirubin and urea). In addition, to further confirm the effect of cells in the bioreactor system, models were connected to acellular bioreactors and a significant difference in parameters observed, eliminating the possibility of any adsorption effects of the scaffold. Bilirubin showed a significant decrease (approximately 60%; 2.30 ± 0.25 mg/dL to 0.95 ± 0.01 mg/dL) (Fig. 5A), whereas AST reduced by 20% (353.4 ± 7.2 to 280.6 ± 7.4 U/L) in models connected to the cell-laden bioreactor (Fig. 5B). Also, urea showed a significant increase from 10.9 ± 0.7 mg/dL to 15.3 ± 0.14 mg/dL (approximately 40%) (Fig. 5C). Ammonia levels in the models connected to cell-seeded bioreactors showed a gradual decrease (18%) (Fig. 5D) and remained lower than those in models not connected to the bioreactor. However, the levels did not change much beyond the first 90 minutes of connection. This could be attributed to the lack of sufficient ammonia metabolism by HepG2 cells^36^,^37^. Interestingly, models connected to acellular bioreactors showed significantly lower values for bilirubin, AST and ammonia as compared to untreated models (Fig. 5A,B and D) confirming the efficacy of the activated carbon cloth as a detoxifying unit.

Albumin levels in models connected to the cell-laden bioreactor showed a mere 6% increase (2.39 ± 0.14 g/dL to 2.54 ± 0.12 g/dL) (Fig. 5E). To confirm the functionality of the cell-loaded bioreactor, human albumin was quantified in rat plasma. Healthy rats connected to the bioreactor showed higher amounts of human albumin in plasma (approx. 21.0 ± 0.8 ng/mL) compared to models (approx. 19.5 ± 0.7 ng/mL) (Fig. 5F). This could be due to the detrimental effect of the plasma which, though clarified to some extent by the activated carbon cloth, may still have some toxic effects.

**Figure 6:**
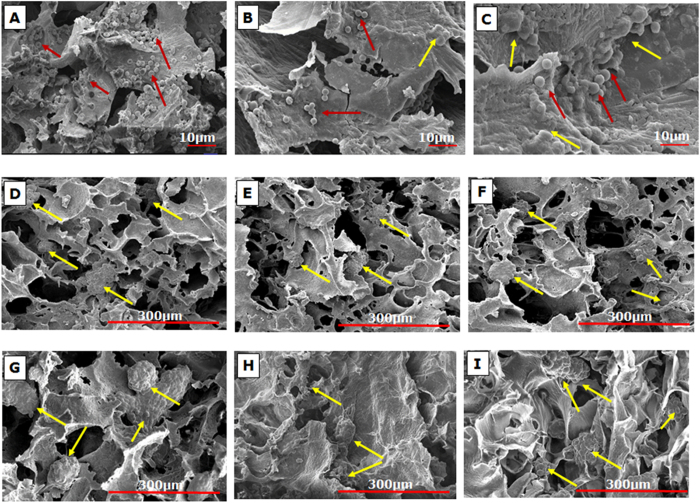
Scanning electron micrographs of cryogel bioreactors. SEM images of the top (**A**), middle (**B**) and bottom (**C**) section of the monolith cryogel bioreactor used in System 1 at the end of extracorporeal run showing blood cell infiltration (red arrows); Negligible cell infiltration was observed in the left (**D**), middle (**E**) and right (**F**) sections of the cryogel disc used in System 2; Micrographs of the left (**G**), middle (**H**) and right (**I**) sections of unused cell-seeded bioreactor show spheroid formation of the hepatic cells (yellow arrows) on the cryogel matrix.

Scanning electron micrographs of the reactor on completion of animal study showed infiltration of blood cells into the bioreactor from System 1 (Fig. 6A–C). Ideally, the use of whole blood in a BAL system would be advantageous since erythrocytes may act as potential oxygen delivery vehicles for the cells housed in the bioreactor^38^. However leukocyte activation and cell damage limits the application of whole blood in BAL^38^. Inherent limitations of improper sealing of the lab-scale plasma separators devised from packing hollow fiber plasma separation membranes in a tubular module resulted in their loss of efficiency of plasma separation, leading to leakage of blood cells into the bioreactor. This could have further aggravated cell damage initiated by toxins in the plasma hence reducing the efficacy of the bioreactor system. Further, scanning electron micrographs of the cryogel reactor from System 2 showed negligible blood cell infiltration due to the use of the plasma separation membrane in a sheet format that fit well with the bioreactor design, preventing any form of leakage, though the hepatic cells in the reactor look smaller and less rounded (Fig. 6D–F) in comparison to cells in the unused cryogel reactor (Fig. 6G–I). This change in morphology may be attributed to the toxicity of the plasma which, though reduced, may still have detrimental effects.

### Offline evaluation of the integrated cryogel bioreactor

To further corroborate the results from extracorporeal connection of the integrated bioreactor, patient plasma collected for routine clinical testing was perfused through the integrated cryogel based bioreactor for a period of 3 h. The average level of bilirubin was reduced by up-to 70.4 ± 5.8% after treatment. Similarly, ammonia levels and AST levels reduced by 39.8 ± 4.6% and 37.5 ± 20%, respectively while albumin and urea increased by 60.6 ± 25% and 25.3 ± 24%, respectively (Table 1). These results indicate the ability of the integrated bioreactor to perform both detoxification as well as synthetic functions effectively. On comparison with the offline evaluation of patient plasma using the monolith bioreactor^20^, it was found that the integrated cryogel based bioreactor shows better efficacy in terms of detoxification of bilirubin and ammonia and production of albumin in patient plasma. This could be attributed to the presence of the activated carbon cloth in the integrated bioreactor which removes some of the toxins that may limit the function of the cells present in the cryogel based bioreactor.

## Conclusion

An integrated hybrid cryogel based bioreactor was developed that showed better efficacy in terms of detoxification as well as synthetic functions than the conventional BAL setup. Integrating the system resulted in reduced volume of blood withdrawn from animals (6–8 mL in System 1 vs. 4–6 mL in System 2). However, there is still a limitation regarding exposure of cells in the bioreactor to toxins in the plasma. Although activated carbon reduces the toxin load to some extent, plasma still poses a threat to the cells in the bioreactor.

## Additional Information

**How to cite this article**: Damania, A. *et al*. Alleviating liver failure conditions using an integrated hybrid cryogel based cellular bioreactor as a bioartificial liver support. *Sci. Rep.* **7**, 40323; doi: 10.1038/srep40323 (2017).

**Publisher's note:** Springer Nature remains neutral with regard to jurisdictional claims in published maps and institutional affiliations.

## Figures and Tables

**Table 1:** Offline evaluation of integrated hybrid cryogel based bioreactor seeded with HepG2 cells.

	Bilirubin			AST			Ammonia			Albumin			Urea		
	UT	T	% change	UT	T	% change	UT	T	% change	UT	T	% change	UT	T	% change
P1	15.09	3.86	−74.42	341.2	105.9	−68.96	290.3	189.5	−34.72	2.38	3.59	+50.84	7.12	12.66	+77.81
P2	12.51	3.37	−73.06	201.6	105.3	−47.77	266.1	163.9	−38.41	1.68	3.36	+100	9.59	12.66	+32.01
P3	3.91	0.99	−74.68	68.95	58.34	−15.39	276.2	162.3	−41.24	2.01	3.13	+55.72	12.33	12.81	+3.89
P4	8.04	3.12	−61.19	208.6	85.3	−59.11	282.6	166.3	−41.15	2.22	3.74	+68.47	9.86	12.81	+29.9
P5	6.84	1.99	−70.91	118.5	107.4	−9.37	289.5	200.7	−30.67	1.76	3.31	+88.07	10.55	10.45	−0.95
P6	3.9	1.09	−72.05	150.3	82.2	−45.31	293.3	164.4	−43.95	1.75	3.61	+31.27	7.31	9.63	+31.74
P7	3.3	1.33	−59.7	178.6	102.3	−42.72	263.9	146.3	−44.56	2.04	3.42	+67.65	12.33	12.35	+0.162
P8	3.85	1.05	−72.72	111.4	80	−28.19	272.4	153.1	−43.8	1.94	3.16	+62.89	7.81	9.5	+21.64
P9	5.27	1.34	−74.57	106.1	84.2	−20.64	275.2	165.2	39.97	1.51	3.03	+20.72	7.31	9.62	+31.6
Avg	−70.4 ± 5.8			−37.5 ± 20			−39.8 ± 4.6			+60.6 ± 25			+25.3 ± 24		
Ref	0.3–1.2 mg/dL			10–40 IU/L			12–60 μg/dL			3.5–5.2 g/dL			15–40 mg/dL		

UT: Untreated patient plasma; T: Treated patient plasma. Percent change in parameter post-treatment calculated as the difference between T and UT divided by UT multiplied by 100 (i.e ((T-UT)/UT)*100). Positive (negative) sign represents an increase (decrease). Average (Avg) is the mean ± SD (n = 9) value of percent increase or decrease obtained from 9 samples treated in the integrated bioreactor. Reference (Ref) is the normal human plasma values for the biochemical parameters in the table.

## References

[b1] Lee K. C. L., Stadlbauer V. & Jalan R. Extracorporeal Liver Support Devices for Listed Patients. Liver Transpl, doi: 10.1002/lt.24396 (2016). 26785141

[b2] Jalan R., Sen S. & Williams R. Prospects For Extracorporeal Liver Support. Gut 53, 890–898, doi: 10.1136/gut.2003.024919 (2004). 15138219 PMC1774070

[b3] Aron J., Agarwal B. & Davenport A. Extracorporeal support for patients with acute and acute on chronic liver failure. Expert Review of Medical Devices, 1–14, doi: 10.1586/17434440.2016.1154455 (2016). 26894968

[b4] Bernal W., Auzinger G., Dhawan A. & Wendon J. Acute liver failure. The Lancet 376, 190–201, doi: 10.1016/S0140-6736(10)60274-7. 20638564

[b5] Bernal W. . Acute-on-chronic liver failure. The Lancet 386, 1576–1587, doi: 10.1016/S0140-6736(15)00309-8. 26423181

[b6] Stange J. Extracorporeal liver support. Organogenesis 7, 64–73, doi: 10.4161/org.7.1.14069 (2011). 21343699 PMC3082035

[b7] Carpentier B., Gautier A. & Legallais C. Artificial and bioartificial liver devices: present and future. Gut 58, 1690–1702, doi: 10.1136/gut.2008.175380 (2009). 19923348

[b8] Pless G. Artificial and Bioartificial Liver Support. Organogenesis 3, 20–24 (2007). 19279696 10.4161/org.3.1.3635 PMC2649611

[b9] Riordan S. & Williams R. Bioartificial liver support: developments in hepatocyte culture and bioreactor design. British Medical Bulletin 53, 730–744 (1997). 9536524 10.1093/oxfordjournals.bmb.a011644

[b10] Pless G. In Hepatocytes Vol. 640 *Methods in Molecular Biology* (ed Patrick Maurel) Ch. 28, 511-523 (Humana Press, 2010).

[b11] Bañares R. . Extracorporeal albumin dialysis with the molecular adsorbent recirculating system in acute-on-chronic liver failure: The RELIEF trial. Hepatology 57, 1153–1162, doi: 10.1002/hep.26185 (2013). 23213075

[b12] Saliba F. . Albumin Dialysis With a Noncell Artificial Liver Support Device in Patients With Acute Liver FailureA Randomized, Controlled Trial. Annals of Internal Medicine 159, 522–531, doi: 10.7326/0003-4819-159-8-201310150-00005 (2013). 24126646

[b13] Lexmond W. S. . Experience with molecular adsorbent recirculating system treatment in 20 children listed for high-urgency liver transplantation. Liver Transplantation 21, 369–380, doi: 10.1002/lt.24037 (2015). 25366362

[b14] Sussman N. L. & Kelly J. H. Artificial Liver. Clinical Gastroenterology and Hepatology 12, 1439–1442, doi: 10.1016/j.cgh.2014.06.002 (2014). 24909908

[b15] Zeilinger K. & Gerlach J. C. Artificial Liver Bioreactor Design. Bioreactor Design: Design, Operation and Novel Applications (2016).

[b16] Mullon C. & Pitkin Z. The HepatAssist® Bioartificial Liver Support System: clinical study and pig hepatocyte process. Expert opinion on investigational drugs 8, 229–235 (1999). 15992074 10.1517/13543784.8.3.229

[b17] Demetriou A. A. . Prospective, randomized, multicenter, controlled trial of a bioartificial liver in treating acute liver failure. Annals of surgery 239, 660 (2004). 15082970 10.1097/01.sla.0000124298.74199.e5 PMC1356274

[b18] Shi X.-L. . Evaluation of a novel hybrid bioartificial liver based on a multi-layer flat-plate bioreactor. World Journal of Gastroenterolog: WJG 18, 3752–3760, doi: 10.3748/wjg.v18.i28.3752 (2012). PMC3406430 22851870

[b19] Han B. . No transmission of porcine endogenous retrovirus in an acute liver failure model treated by a novel hybrid bioartificial liver containing porcine hepatocytes. Hepatobiliary & Pancreatic Diseases International 14, 492–501, doi: 10.1016/S1499-3872(15)60401-5 (2015). 26459725

[b20] Jain E. . Fabrication of macroporous cryogels as potential hepatocyte carriers for bioartificial liver support. Colloids and Surfaces B: Biointerfaces 136, 761–771 (2015). 26519938 10.1016/j.colsurfb.2015.10.012

[b21] Jain E. & Kumar A. 15 Cryogel Bioreactor for Therapeutic Protein Production. Supermacroporous Cryogels: Biomedical and Biotechnological Applications, 419 (2016).

[b22] Flendrig L. M. . Evaluation of a novel bioartificial liver in rats with complete liver ischemia: treatment efficacy and species-specific α-GST detection to monitor hepatocyte viability. Journal of Hepatology 30, 311–320, doi: 10.1016/S0168-8278(99)80078-6 (1999). 10068112

[b23] Park J.-k. & Lee D.-h. Bioartificial liver systems: current status and future perspective. Journal of bioscience and bioengineering 99, 311–319 (2005). 16233796 10.1263/jbb.99.311

[b24] Riordan S. M. & Williams R. Extracorporeal support and hepatocyte transplantation in acute liver failure and cirrhosis. Journal of Gastroenterology and Hepatology 14, 757–770, doi: 10.1046/j.1440-1746.1999.01945.x (1999). 10482426

[b25] Sekas G. & Cook R. T. The evaluation of liver function after partial hepatectomy in the rat: serum changes. British journal of experimental pathology 60, 447–452 (1979). 518814 PMC2041499

[b26] Ash S. R., Sullivan T. A. & Carr D. J. Sorbent Suspensions vs. Sorbent Columns for Extracorporeal Detoxification in Hepatic Failure. Therapeutic Apheresis and Dialysis 10, 145–153, doi: 10.1111/j.1744-9987.2006.00356.x (2006). 16684216

[b27] Müller B. R. Effect of particle size and surface area on the adsorption of albumin-bonded bilirubin on activated carbon. Carbon 48, 3607–3615, doi: 10.1016/j.carbon.2010.06.011 (2010).

[b28] Nibourg G. A. . Effects of acute‐liver‐failure‐plasma exposure on hepatic functionality of HepaRG‐AMC‐Bioartificial Liver. Liver International 33, 516–524 (2013). 23387413 10.1111/liv.12090

[b29] Hughes R., Cochrane A., Thomson A., Murray-Lyon I. & Williams R. The cytotoxicity of plasma from patients with acute hepatic failure to isolated rabbit hepatocytes. British journal of experimental pathology 57, 348 (1976). 986149 PMC2041069

[b30] Smirthwaite A. D., Gaylor J. D., Cousins R. B. & Grant H. Cytotoxicity of bile in human Hep G2 cells and in primary cultures of rat hepatocytes. Artificial organs 22, 831–836 (1998). 9790080 10.1046/j.1525-1594.1998.06088.x

[b31] Martins P. N., Theruvath T. P. & Neuhaus P. Rodent models of partial hepatectomies*. Liver International 28, 3–11 (2008). 18028319 10.1111/j.1478-3231.2007.01628.x

[b32] Pringle J. H. V. Notes on the Arrest of Hepatic Hemorrhage Due to Trauma. Annals of Surgery 48, 541–549 (1908). 17862242 10.1097/00000658-190810000-00005 PMC1406963

[b33] Dixon E., Vollmer C. M. Jr, Bathe O. F. & Sutherland F. Vascular occlusion to decrease blood loss during hepatic resection. The American Journal of Surgery 190, 75–86, doi: 10.1016/j.amjsurg.2004.10.007 (2005). 15972177

[b34] Selzner M., Camargo C. A. & Clavien P.-A. Ischemia impairs liver regeneration after major tissue loss in rodents: Protective effects of interleukin-6. Hepatology 30, 469–475, doi: 10.1002/hep.510300215 (1999). 10421656

[b35] Helling T. S. Liver failure following partial hepatectomy. HPB: The Official Journal of the International Hepato Pancreato Biliary Association 8, 165–174, doi: 10.1080/13651820510035712 (2006). 18333270 PMC2131683

[b36] Enosawa S. . Off-line bioartificial liver: a novel concept of treatment and its potency of liver regeneration. Transplantation Proceedings 34, 2711–2713, doi: 10.1016/S0041-1345(02)03384-5 (2002). 12431583

[b37] Mavri-Damelin D. . Ornithine transcarbamylase and arginase I deficiency are responsible for diminished urea cycle function in the human hepatoblastoma cell line HepG2. The International Journal of Biochemistry & Cell Biology 39, 555–564, doi: 10.1016/j.biocel.2006.10.007 (2007). 17098461

[b38] Allen J. W., Hassanein T. & Bhatia S. N. Advances in bioartificial liver devices. Hepatology 34, 447–455 (2001). 11526528 10.1053/jhep.2001.26753

